# Therapeutic priming of liver regeneration: from mechanisms to *ex vivo* machine perfusion

**DOI:** 10.3389/frtra.2026.1841091

**Published:** 2026-06-24

**Authors:** Sarah Yoshimi Tropp, Dinesh Jaishankar, Carl Atkinson, Satish N. Nadig

**Affiliations:** 1 Comprehensive Transplant Center, Feinberg School of Medicine, Northwestern University, Chicago, IL, United States; 2 Department of Surgery, Feinberg School of Medicine, Northwestern University, Chicago, IL, United States; 3 Department of Microbiology and Immunology and Pediatrics, Northwestern University, Chicago, IL, United States

**Keywords:** allograft, hepatocytes, liver transplant, machine perfusion, regenerative medicine

## Abstract

The liver's extraordinary regenerative capacity has fascinated biologists for decades, yet the translation of regenerative biology into clinically effective therapies remains limited. Regeneration is not a simple proliferative response but a tightly orchestrated program initiated by tissue injury and coordinated through inflammatory priming, complement activation, mechanosensory signaling, extracellular matrix remodeling, metabolic reprogramming, and growth factor–driven hepatocyte proliferation. In this review, we synthesize current understanding of the cellular and molecular networks that govern liver regeneration, emphasizing the dynamic crosstalk among hepatocytes, Kupffer cells, liver sinusoidal endothelial cells, hepatic stellate cells, and recruited immune populations. We further examine the major experimental systems used to study regeneration, from classic *in vivo* hepatectomy models to emerging human-relevant platforms, including organoids, liver-on-a-chip systems, extracellular matrix scaffolds, precision-cut liver slices, and *ex vivo* perfusion, highlighting both their mechanistic value and translational limitations. We then focus on the growing clinical importance of regeneration in transplantation, where the expanding use of marginal, steatotic, aged, and reduced-size grafts has intensified the need to preserve regenerative competence. In this context, *ex vivo* machine perfusion offers a uniquely promising platform to study, modulate, and therapeutically prime regenerative pathways before implantation, potentially enabling graft reconditioning strategies that improve outcomes in small-for-size and other high-risk liver grafts.

## 1 Introduction

The liver has long occupied a singular place in regenerative biology, dating back to the myth of Prometheus, whose eternally regenerating liver symbolized the organ's remarkable restorative capacity. Modern experimental studies have since shown that liver regeneration is not a simple proliferative response, but a tightly coordinated program involving inflammatory priming, complement activation, mechanosensory signaling, extracellular matrix remodeling, metabolic reprogramming, and growth factor–driven hepatocyte proliferation (1). Since the seminal partial hepatectomy (PHx) studies of Higgins and Anderson, a wide range of *in vivo* and *ex vivo* models have defined many of the pathways that govern this response; however, translation of these biological insights into clinically actionable therapies remains limited (2). This challenge has become increasingly important in transplantation, where broader reliance on marginal, steatotic, aged, and reduced-size grafts has heightened the need to preserve not only viability, but also regenerative competence. In this context, machine perfusion has emerged as a particularly promising platform, offering a controllable *ex vivo* system in which whole-organ physiology can be maintained. This platform enables regenerative pathways to be assessed, manipulated, and potentially therapeutically enhanced prior to implantation. In this review, we summarize the core mechanisms that initiate and sustain liver regeneration, examine the experimental models used to study this process, and discuss how *ex vivo* perfusion platforms may help translate regenerative biology into targeted strategies for graft reconditioning and repair.

## 2 Biology of liver regeneration

**Figure 1:**
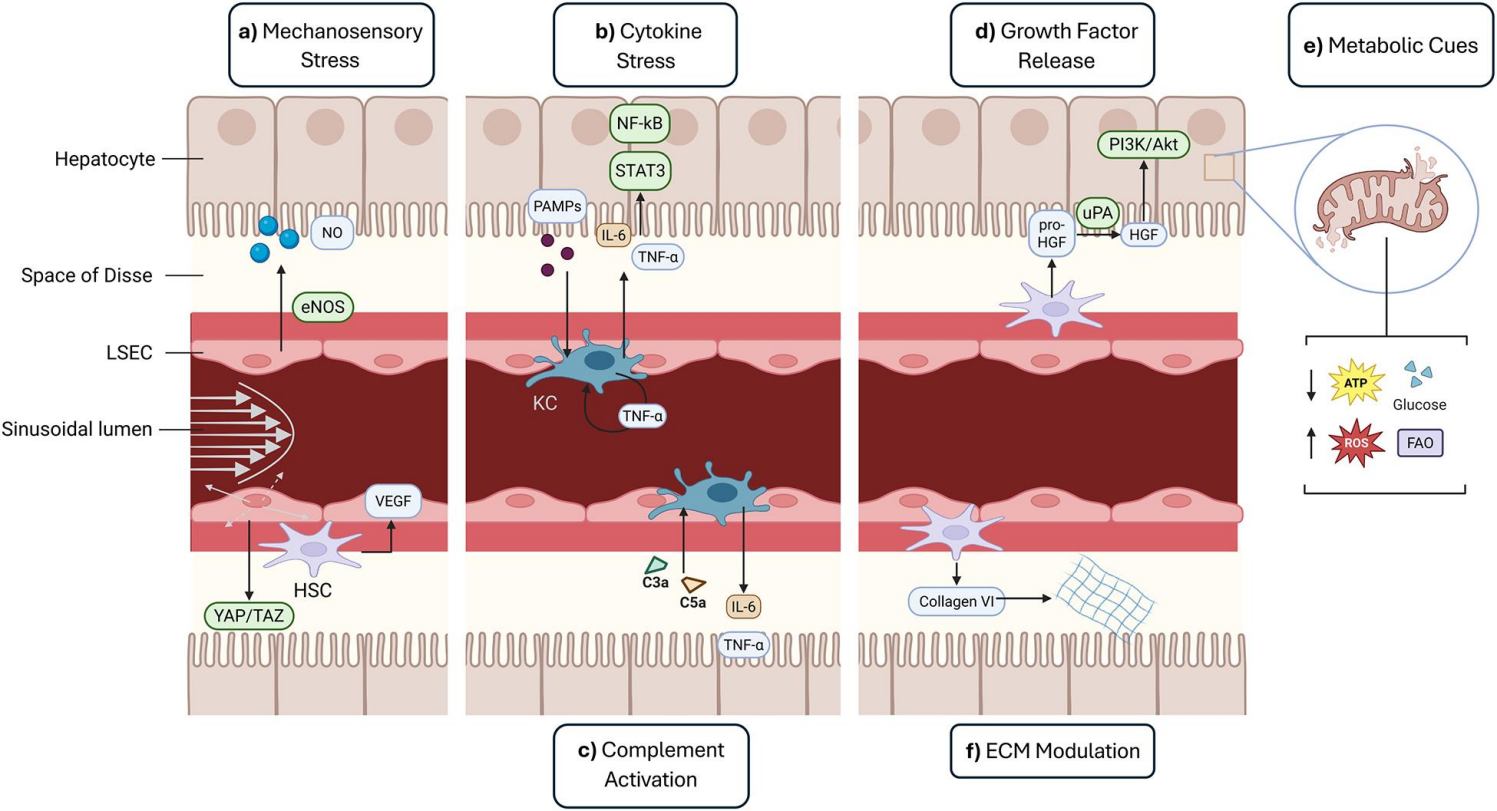
Multicellular signaling mechanisms initiating liver regeneration. Following partial hepatectomy (PHx), the residual hepatic sinusoidal unit, comprising hepatocytes, liver sinusoidal endothelial cells (LSECs), Kupffer cells (KCs), and hepatic stellate cells (HSCs) coordinates a rapid, multi-signal regenerative response. **(a)** Increased portal shear stress activates mechanosensory pathways, including YAP/TAZ, in hepatocytes and LSECs. **(b)** Activated KCs release TNF-α and IL-6, engaging NF-*κ*B and STAT3 transcription factors to prime hepatocytes for proliferation. **(c)** Complement-derived anaphylatoxins C3a/C5a amplify KC activation and cytokine signaling. **(d)** LSECs and HSCs release mitogenic growth factors, including HGF and EGF, which drive hepatocyte cell cycle progression via MET and EGFR signaling. **(e)** Systemic metabolic cues, including bile acids and ATP, provide auxiliary regenerative signals. **(f)** Dynamic ECM remodeling by HSCs facilitates hepatocyte proliferation and restoration of hepatic architecture. Together, these interconnected signals orchestrate the initiation and propagation of liver regeneration. PHx, partial hepatectomy; LSEC, liver sinusoidal endothelial cell; HSC, hepatic stellate cell; KC, Kupffer cell; HGF, hepatocyte growth factor; EGF, epidermal growth factor; ECM, extracellular matrix; YAP/TAZ, Yes-associated protein/transcriptional coactivator with PDZ-binding motif; NF-*κ*B, nuclear factor kappa B; STAT3, signal transducer and activator of transcription 3; TNF-α, tumor necrosis factor alpha. Created in Biorender.

Liver regeneration is initiated within minutes of tissue loss and is facilitated through the rapid integration of inflammatory, metabolic, and mechanosensory signals that collectively prime hepatocytes to re-enter the cell cycle. Although hepatocytes ultimately execute the regenerative program, the initial phase is driven almost entirely by non-parenchymal cells, particularly Kupffer cells (KCs), liver sinusoidal endothelial cells (LSECs), hepatic stellate cells (HSCs), and recruited innate immune populations. Together, these cells sense injury and orchestrate the early signaling milieu that “primes” hepatocytes to transition from a quiescent G0 state into a transcriptionally active, cell-cycle-competent proliferative state. These early regenerative events and temporal integration are discussed in detail below and summarized in Figure 1.

### 2.1 Early injury signals and DAMP sensing

Following hepatocyte injury and cell loss in the setting of sterile inflammation, the liver rapidly releases damage-associated molecular patterns (DAMPs) that activate innate immune pathways and alert multiple cellular compartments within the hepatic microenvironment (3). These endogenous danger signals are detected through pattern recognition receptors (PRRs), which promote the recruitment and activation of resident immune cells, particularly Kupffer cells (KCs) (4). Thus, DAMP sensing represents one of the earliest events that precede regenerative priming (5).

Among these pathways, Toll-like receptor 4 (TLR4) plays a central role. TLR4 activation through high-mobility group box 1 (HMGB1) and members of the S100 protein family, leading to reactive oxygen species (ROS) generation and activation of downstream inflammatory cascades (6). HMGB1 also signals through the receptor for advanced glycation end products (RAGE), further amplifying KC activation (7). S100 proteins, released by multiple cell types during inflammatory stress, are consistently upregulated in both acute and chronic liver inflammation and further contribute to macrophage activation (5). Collectively, these DAMP-associated pathways enable the injured liver to detect cellular damage and generate the proinflammatory signals required for KC priming.

Hepatocyte injury also leads to the release of cell-free mitochondrial DNA and extracellular ATP, both of which amplify sterile inflammation. Mitochondrial DNA engages Toll-like receptor 9 (TLR9) and is also sensed through cyclic GMP-AMP synthase (cGAS), signaling through interferon regulatory factor 3 (IRF3) to induce downstream inflammatory transcriptional programs (8). Extracellular ATP acts as a “find-me” signal released by injured hepatocytes and binds purinergic receptors on neighboring immune cells, promoting NLR family pyrin domain-containing 3 (NLRP3) inflammasome activation through caspase-dependent mechanisms. In turn, NLRP3 activation and mitochondrial dysfunction further accelerate ROS generation and contribute to the activation of NF-*κ*B and MAPK signaling pathways. Together, these signals generate a rapid burst of cytokine production, chemokine secretion, and adhesion molecule expression that establishes the early pro-regenerative inflammatory milieu (9).

Importantly, these early danger signals do not directly drive hepatocyte proliferation. Rather, they function to activate the liver's immune and secretory network, creating the conditions necessary for cytokine-mediated hepatocyte priming. In this context, KCs serve as key early relays that translate tissue injury into the inflammatory signals required to initiate regeneration.

### 2.2 Cytokine-mediated priming of hepatocytes

Following DAMP sensing, KCs release proinflammatory cytokines, in particular, tumor necrosis factor-alpha (TNF*α*) and interleukin-6 (IL-6) (10, 11). The mechanistic importance of TNF*α* and IL-6 has been demonstrated in rodent PHx models. Animals deficient in TNF*α* and IL-6 demonstrate impaired liver regeneration, supporting an important role in promoting hepatocyte proliferation and maintaining cellular survival through inflammatory signaling (12, 13). Release of TNF*α* and IL-6 activates pro-survival pathways through signaling relays such as NF-κB and STAT3 in hepatocytes (Figure 1B).

TNF*α* functions primarily as an early inflammatory priming signal rather than a complete mitogen (14). Through TNF*α* Receptor 1 (TNFR1) dependent signaling, TNF*α* promotes NF-*κ*B activation and supports IL-6 production by Kupffer cells, thereby creating a cytokine environment that licenses hepatocytes to respond to downstream mitogenic cues (15). Subsequent cell-cycle progression is driven by coordinated activation of growth factor pathways, including HGF/c-MET and EGFR signaling, together with PI3K/Akt, Hippo/YAP, and other cell-cycle regulatory programs (12). Further support for the vital role of TNF*α* comes from TNF*α* inhibition rodent study, which revealed that blocking TNF*α* leads to impaired DNA synthesis after PHx (16). Collectively, these studies demonstrate the crucial role of TNF*α* in the propagation of regenerative signals.

Since TNF*α* supports IL-6 production, IL-6 expression follows shortly after, but both peak within 2-7 h post-injury (14). IL-6 supports hepatocyte proliferation by binding to IL-6R/GP130 receptors on hepatocytes, acting as a protective mechanism signaling against injury (17). Activation of gp130 enables STAT3 signaling in hepatocytes to promote pro-survival and anti-apoptotic signaling, preventing further hepatocyte loss. Under physiological conditions, STAT3 signaling is tightly regulated through a negative feedback loop of SOCS3 (Suppressor of Cytokine Signaling 3) production, and degrades cytokine receptors (18). However, an anti-inflammatory and NF-κB inhibitory protein, A20, downregulates SOCS3 to enhance the IL-6/STAT3 pro-proliferative signals in hepatocytes during the priming stage of liver regeneration (19).

### 2.3 Complement activation as an initiator of hepatic regeneration

In recent years, complement activation has emerged as an active regulator of liver regeneration rather than a mere passive bystander in the inflammatory response. Foundational studies established that complement 3 (C3) and complement 5 (C5) are required during the earliest priming phase after PHx or toxic injury, as deficiency of either pathway impairs regeneration, increases parenchymal damage, and worsens survival (20–23) (Figure 1C). Mechanistically, the principal pro-regenerative complement effectors are C3a and C5a, which signal through C3a receptor (C3aR) and C5a receptor 1 (C5aR1) and C5aR2 to promote Kupffer cell production of TNF*α* and IL-6, thereby driving NF-*κ*B and STAT3 activation in hepatocytes that ultimately promotes their entry into the cell cycle (24). In addition to promoting cell cycle initiation, signaling of C3aR and C5aR has been demonstrated to be essential for minimizing apoptotic cell loss. Studies with C3aR and C5aR-deficient mice showed that deficiency was associated with prominent hepatocyte apoptosis during the regeneration phase in a model of liver hepatectomy, indicating that complement also provides a cytoprotective signal that supports survival of the regenerating parenchyma. Notably, reconstitution experiments suggested that optimal rescue requires both C3a and C5a, consistent with a cooperative model in which these anaphylatoxins jointly establish the cytokine milieu necessary for effective regeneration and tissue preservation. More recent work has expanded on this framework, beyond inflammatory priming alone. Analysis of the liver regeneration priming phase in C3-deficient mice indicates that complement-dependent regeneration is coupled with induction of immediate-early response programs, including c-fos, as well as activation of acute-phase pathways and remodeling of sterol and cholesterol metabolism. These observations suggest that complement helps synchronize inflammatory signaling with the metabolic adaptation required for regenerative growth, an important concept given the biosynthetic demands placed on hepatocytes as they re-enter the cell cycle. Additional studies further indicate that C5aR signaling intersects with HGF/c-MET–associated regenerative pathways, while noncanonical plasmin-mediated C3 activation may also provide an additional route for generating pro-regenerative complement signals in the injured liver. Together, these data position complement as a coordinator of inflammatory, survival, and metabolic programs during the earliest phases of liver regrowth.

Interestingly, the regenerative benefit of complement activation is dose- and context-dependent. Studies using site-targeted complement inhibitors emphasize a threshold model in which controlled upstream C3/C5 activation supports recovery, whereas excessive downstream complement activation exacerbates hepatocellular injury. This distinction is particularly important for the membrane attack complex, which contributes disproportionately to tissue damage. In support of this concept, targeted inhibition of terminal complement with CR2-CD59 improved regeneration and long-term survival after severe hepatectomy, while preserving and enhancing hepatic TNF*α*, IL-6, STAT3, and Akt signaling, reducing mitochondrial depolarization, and facilitating recovery of ATP stores (25). Other studies likewise show that complete C3 deficiency or broad C3 inhibition is detrimental, whereas partial modulation can preserve beneficial IL-6/STAT3 signaling, limit oxidative stress, and improve hepatocyte proliferation. Through the tempering of terminal pathway activation, it was discovered that C3adesArg, a metabolite of C3a, acting through C5aR2, was a primary signaling conduit for improved regeneration (26).

Collectively, these findings support a more integrated model in which complement links innate immune sensing to regenerative execution. By shaping KC cytokine release, sustaining hepatocyte survival pathways, engaging acute-phase and immediate-early transcriptional programs, and facilitating metabolic reprogramming, complement helps convert tissue loss into a coordinated regenerative response. This perspective also helps explain why complement-targeted therapies in the liver must be precisely tuned strategies that blunt injurious terminal activation, while preserving early C3/C5-derived regenerative signals may be more effective than indiscriminate blockade of the cascade.

### 2.4 Hemodynamic and mechanical signaling

Priming for liver regeneration is critically influenced by mechanical forces detected within the first 5 min of tissue loss. Vascular architecture of the liver is uniquely structured with high-pressure arterial inflow (around 90 mmHg) and low-pressure portal venous inflow (between 3 and 10 mmHg) converging within hepatic sinusoids (27). Arterial and portal flow into the central vein creates a pressure decline from the periportal to pericentral region, decreasing to 1–5 mmHg. Under physiological conditions, the low blood pressure and low flow velocity facilitate the exchange of oxygen, nutrients, and toxins between the circulating blood and hepatocytes (28). With tissue loss, blood flow can increase in small regions of the liver, influencing pressure dynamics and mechanosensing mechanisms.

Early observations linked the increase of blood flow to compensatory liver growth gave rise to the “blood-flow theory” of liver regeneration, where hemodynamic forces act as active signals rather than passive consequences of tissue loss (29). Blood-flow theory links pressure dynamics, influenced by vasculature resistance, to active regulation of metabolic and nutrient signaling in the liver sinusoid. The onset of ischemic liver injury alters hemodynamics through vessel dilation, an increase in portal flow, and an increase in shear stress within the sinusoidal network (28). The change in hemodynamics permits liver sinusoidal endothelial cells (LSECs) and hepatic stellate cells (HSCs) to undergo mechanical stress, which alters intracellular signaling impacted by changes in oxygen and nutrient supply to the liver sinusoid, specifically through nitric oxide (NO) production (Figure 1A).

#### 2.4.1 LSEC and HSC mechanosensing

Ischemic liver injury increases shear stress, detected by LSECs as the primary mechanosensors, decreasing cell-to-cell contact to mechanically expand intracellular spacing (30). Shear stress permits the activation of ion channels, resulting in Ca^2+^ influx and induction of KLF2 (Kruppel-like factor 2) (31). KLF2 acts as a mechanosensitive transcription factor that drives the expression of NO production and endothelial NO synthase (eNOS) production.HSCs, located within the space of Disse, are also exposed to increased flow and pressure due to the fenestrated nature of the sinusoidal endothelium (32). An increase in laminar and pulsatile flow presents an increase in HGF secretion and production by HSCs while reducing sequestration of inactive, matrix-bound HGF (pro-HGF) through *α*5/*β*1 integrin-mediated mechanosensing (33). Both these detections permit the release of priming signals for liver regeneration.

#### 2.4.2 Nitric oxide (NO) production

Mechanical stimulation of LSECs induces endothelial nitric oxide synthase (eNOS) in LSECs and inducible NOS (iNOS) in KCs. NO acts as a vasodilator, influencing the permeability of growth factors and facilitating delivery into hepatocytes. Hepatic iNOS is induced by inflammatory cytokines in KCs, further upregulating NO generation. Previous research has found that NO plays an important role in balancing apoptosis and proliferation, as it exerts context-dependent effects on hepatocyte survival and proliferation (34).

NO also plays a well-characterized role in angiogenesis and vascular remodeling by increasing vascular endothelial growth factor (VEGF) levels. Activation of VEGF Receptor 2 (VEGFR-2) triggers VEGF Id1-dependent release of hepatocyte growth factor (HGF) and Wnt2, potent hepatocyte proliferative and angiogenic factors (35, 36). In LSECs, VEGFR-2 acts as a mechanosensor by responding to laminar shear stress and translocating from the perinuclear membrane to cytoskeletal localization, thus driving angiogenesis through the VEGFR2-Id1-Wnt signaling axis (37).

#### 2.4.3 Cyclic stretch

Hemodynamic and biomechanical changes that follow liver injury constitute an important layer of regenerative signaling. Increases in sinusoidal flow and intrahepatic pressure expose LSECs to cyclic stretch, which is sensed through mechanotransducive pathways involving *β*1 integrin, VEGFR-3, and G protein-coupled receptors. These inputs promote Ca^2+^ influx and ERK activation and are further reinforced by inflammatory cytokines such as IL-6 and TNF*α* (38, 39). In response, LSECs release angiocrine and paracrine mediators, including HGF, Wnt2, NO, IL-6, and TNF*α*, thereby coupling altered vascular forces to hepatocyte priming, proliferative signaling, and sinusoidal adaptation.

HSCs likewise respond dynamically to mechanical stress generated by increased intrahepatic pressure and evolving matrix stiffness. Cyclic stretch activates HSCs and induces matrix metalloproteinase-1 (MMP-1), supporting the concept that these cells participate early in pressure-sensitive extracellular matrix remodeling (27). Beyond MMP-1, activated HSCs contribute to the regenerative niche by secreting HGF, VEGF, cytokines, and matrix components that reshape the local microenvironment and facilitate coordinated tissue expansion. In parallel, mechanical cues converge on YAP/TAZ signaling, linking cytoskeletal tension to transcriptional programs that promote hepatocyte proliferation (40). Collectively, these findings position LSECs and HSCs as key mechanosensitive effectors of liver regeneration, translating changes in flow, pressure, and matrix stiffness into growth factor release, cytokine signaling, and extracellular matrix remodeling that support regenerative outgrowth.

### 2.5 Microenvironment and the extracellular matrix (ECM)

After parenchymal liver tissue loss, the ECM degrades immediately and needs to remodel to facilitate regeneration, which is normally supported by HSCs (41). The extracellular matrix (ECM) plays a major role in the liver by modulating the binding and release of growth factors to drive proliferation. HSCs receive signals from the ECM through their expression of collagen receptors, integrins, and many other cell-surface protein receptors to modulate the regenerative response (Figure 1F).

Following a PHx, matrix metalloproteinases, including MMP-9, drive ECM degradation, which paradoxically promotes growth factor mobilization (42). Growth factor release activates HSCs, which in turn activate the Hedgehog signaling pathway, the results of which are increased expression of ECM markers, such as increased *α*-SMA expression and collagen production/deposition (43, 44). Further, activated HSCs become producers of pro-HGF via Rho, MTOR, and Hedgehog pathways (45–47). Degradation of ECM as a result of injury provides physical space for pro-HGF to travel across the space of Disse into hepatocytes, where it is converted into its active dimeric form, HGF, through the urokinase (uPA) pathway (48, 49) (Figure 1D).

In summary, following parenchymal tissue loss, rapid ECM degradation and subsequent remodeling create both the structural and signaling conditions required for liver regeneration, with HSCs sensing matrix disruption and responds mobilizing growth factors, activating Hedgehog- and Rho/mTOR-dependent programs, and producing pro-HGF, which is then converted through the uPA pathway into active HGF to drive hepatocyte proliferation.

### 2.6 Metabolic stress as a regenerative cue

Liver regeneration imposes a substantial bioenergetic burden on the remnant parenchyma, and this early regenerative phase is characterized by a marked decline in hepatic ATP levels (Figure 1E). This energetic deficit reflects the abrupt increase in anabolic demand required to support cell growth, macromolecular synthesis, and the broader metabolic adaptations that permit hepatocytes to re-enter the cell cycle (50). To meet these demands, regenerating hepatocytes undergo coordinated metabolic reprogramming, with fatty acid oxidation serving as a major energy source (51). Following PHx, systemic hypoglycemia promotes mobilization of free fatty acids into circulation, contributing to a transient accumulation of hepatic lipids during the early regenerative response (52, 53). This phenomenon, often termed transient regeneration-associated steatosis, is not merely a passive consequence of injury, but likely represents a functional reservoir of metabolic substrates that can be used to generate ATP and provide lipid building blocks for membrane biogenesis during tissue regrowth. However, this process must remain tightly regulated, as defective fatty acid oxidation leads to excessive lipid accumulation and is associated with impaired regenerative signaling.

Glucose metabolism is also dynamically reprogrammed during regeneration. PHx acutely reduces hepatic gluconeogenic capacity, thereby contributing to a transient hypoglycemic state. Notably, exogenous glucose supplementation after hepatectomy has been shown to impair regeneration, at least in part through induction of the cell-cycle inhibitors p21 and p27 (54). These observations suggest that the regenerating liver does not simply require substrate abundance but instead depends on a specific metabolic state that favors proliferative signaling. Consistent with this concept, hepatocytes shift from glucose production to increase glucose utilization via glycolysis to support biosynthetic repair and proliferation. This metabolic transition is reinforced by inflammatory and stress-responsive pathways, including HIF-1*α* signaling, which helps couple the local injury environment to glycolytic reprogramming.

Mitochondrial oxidative stress and amino acid metabolism are linked to building regenerative capacity. One study suggests an increase in mitochondrial oxidation and NADH production correlates with an increase in amino acid metabolism, where pyruvate converts to alanine, and a-ketoglutarate increases cell growth (55). NADH production correlated with enhanced cell-cycle entry and reduced mitochondrial oxidation, while AMP-dependent kinase (AMPK) served as a metabolic checkpoint to coordinate adaptation to hypoxia, ischemia, oxidative stress, and other injuries (56). APMK activation has been demonstrated to reduce lactate accumulation in liver tissue, limiting hepatocyte necrosis during early liver regeneration (57).

Together, these findings indicate that successful liver regeneration requires precise metabolic coordination, in which ATP depletion, fatty acid utilization, transient steatosis, and enhanced glycolysis are integrated to support the energetic and biosynthetic demands of hepatocyte proliferation.

### 2.7 Integration of signals and transition to the proliferative phase

The transition from priming to proliferative hepatocytes is a dynamic and temporally controlled process. Cytokine-mediated STAT3 licenses anti-apoptotic and pro-survival signals in hepatocytes, while Hippo/YAP and PI3 K/Akt signaling pathways drive cell-cycle progression (58).

The Hippo pathway plays a critical role in regulating liver size and development, signaled through the YAP transcription factor. Pharmacological targeting of the Hippo pathway and overexpression of YAP have led to the promotion of liver regeneration and decreased apoptosis (59, 60). Additionally, the PI3 K/Akt pathway plays a key role in regulating the cell cycle and communicates with multiple intracellular pathways around metabolism and cell growth. Multiple studies in various animal models have found that inhibiting or damaging the PI3 K/Akt signaling axis led to impaired liver regeneration (61–63).

Complete mitogens, including HGF and epidermal growth factor (EGF), act as the main drivers of hepatocyte proliferation and cell cycle activation (64, 65). In hepatocytes, HGF binds to c-Met receptor to trigger downstream pathways such as the ERK pathway for hepatocyte proliferation and PI3 K/Akt for pro-survival signals (66). EGF and HB-EGF (heparin-binding EGF), secreted by Brunner's glands, LSECs, and KCs, respectively, bind to EGFR (EGF receptor) on hepatocytes to sustain proliferative responses (67). The multiple family of ligands for EGFR ensures the receptor remains active to sustain proliferative responses via Akt signaling (68).

Auxiliary mitogens, such as bile acids, VEGF, and serotonin, are not independently mitogenic in cultured hepatocytes, but liver regeneration may be delayed in their absence. These mitogens aid in the secretion of HGF and EGF to optimize regenerative efficiency, particularly under conditions of structural or metabolic stress (69).

Liver repair and regeneration merge the connection of inflammatory priming, complement activation, mechanical forces, metabolic adaptations, and growth factor signaling in a dynamically shifting microenvironment. Each of these processes spans from minutes to days and involves interactions between multiple cell populations to prime hepatocytes to acquire proliferative capacity. To uncover this complexity, multiple experimental platforms and injury models have been designed to explore liver regeneration across various scales and to enable controlled manipulation of priming cues while preserving tissue-level architecture and function.

## 3 Models and approaches to study liver regeneration

The modern study of liver regeneration began with the classic rat partial hepatectomy (PHx) model described by Higgins and Anderson in 1931 (2). Rodent models have since been central to defining the kinetics of regeneration and enabling biochemical, functional, and survival-based analyses (70). However, important anatomical and physiological differences limit their ability to fully recapitulate human liver regeneration. This has led to the development of complementary systems, including mouse models, organoids, and precision-cut liver slices, each of which captures distinct aspects of the regenerative response.

Together, these models have provided important insights into the signaling pathways, cellular interactions, biomechanical inputs, and metabolic adaptations that drive liver regrowth. However, because regeneration is a highly coordinated, time-dependent, whole-organ process, no single model fully captures its clinical complexity. This remains a major challenge for translating regenerative biology into clinically relevant therapeutic strategies.

### 3.1 Experimental regeneration through *in vivo* models

PHx remains the canonical *in vivo* model for studying liver regeneration and has provided much of the mechanistic framework that underpins the field. In rodents, PHx enables precise investigation of regenerative kinetics, cytokine release, restoration of liver mass, hepatic function, and survival outcomes. More specialized strains, including mice with humanized liver features, altered immune systems, or targeted gene deletions, have further expanded the ability to model discrete aspects of human liver biology and regenerative signaling. In parallel, toxin-induced injury models, including carbon tetrachloride (CCl_4_), D-galactosamine, acetaminophen, and thioacetamide, have been widely used to study regeneration in the setting of hepatocellular injury rather than tissue resection (71, 72). These systems are especially useful for examining how inflammation, cell death, stellate cell activation, and matrix remodeling shape the regenerative response; for example, CCl_4_ injury has been used to define a role for hepatic stellate cell-derived collagen I in supporting hepatocyte proliferation (73). More broadly, *in vivo* genetic and injury-based models have helped clarify the transcriptional, chromatin, and epigenetic programs that regulate regeneration.

Zebrafish provides a complementary vertebrate model for studying regenerative biology, induced by one-third PHx or targeted hepatic injury (74). Due to their small size, optical transparency, and rapid development, zebrafish are particularly well suited for live imaging, lineage tracing, and fluorescent analysis of regenerative dynamics (75, 76). They have proven especially useful for dissecting conserved developmental and regenerative pathways, including Wnt, BMP, and FGF signaling (77). Their experimental tractability also makes them attractive for rapid pathway discovery and chemical screening.

Despite their major contributions, no *in vivo* model fully reproduces human liver regeneration. Rodents differ substantially from humans in liver architecture, metabolic rate, injury responses, and the tempo of regeneration. For example, rodent hepatocytes exhibit faster metabolism than human hepatocytes, and murine models often fail to capture key features of chronic human liver disease, including hepatocyte senescence (78, 79). Zebrafish, while powerful for mechanistic discovery, are more developmentally oriented and may not fully model adult mammalian regenerative responses. In addition, differences among models in injury magnitude, regenerative timeline, and experimental endpoints complicate cross-study comparisons and translational interpretation. Although the core regenerative pathways are broadly conserved, species-specific differences in anatomy, microenvironment, and physiology remain important limitations when extrapolating *in vivo* findings to human liver regeneration. Thus, the development of human liver regenerative systems is needed urgently to help inform clinical therapeutic translation.

### 3.2 Multicellular coordination and zonation

Since liver regeneration relies on a network of signaling pathways and numerous cytokines and growth factors, the complex process is often studied *in vitro* systems such as 2D cell culture or organoids. Isolated primary hepatocytes are notably difficult to grow and maintain their function in a petri dish, making it difficult to discern long-term regeneration dynamics after injury. Hence, many studies focus on improving *in vitro* systems for growing primary hepatocytes.

Stem cells have a unique capacity for self-renewal, making them an ideal candidate for differentiating into hepatocyte-like cells. The rise in the use of induced pluripotent stem cells and embryonic stem cells opened an avenue for reprogramming cells with growth factors to become hepatocytes. Several studies have shown success in hepatocyte differentiation from embryonic stem cells and in mimicking *in vivo* liver development and function (80–83). One such study found that hepatocyte-like cells derived from pluripotent stem cells could be utilized for regenerative therapy in rats with acute liver failure (84).

Yet, stem cells have their own challenges in fully recapitulating liver regeneration and mature hepatocyte functionality, such that post-differentiation tends to mimic fetal or neonatal phenotypes rather than fully matured hepatocytes (85). Self-replicating hepatocytes still remains a challenge due to heterogeneous expression dictated by zonation, which creates oxygen and metabolomic gradients relative to proximity to the central or portal veins. For example, a prior study found that pericentral hepatocytes have higher Wnt signaling than periportal hepatocytes (86). This zonation and full hepatocyte architecture are difficult to represent in a 2D culture environment.

To improve the architectural model of hepatocytes for measuring liver regeneration, several studies have conducted 2D co-cultures or 3D reconstruction of liver microanatomy. Some studies have opted for co-culturing hepatocytes with other key liver organ cell types, such as LSECs or HSCs, to examine the support needed for hepatocyte proliferation and function (see early sections) (87). These co-culture systems improve albumin production and metabolic hepatocyte activity, further improving the modeling of hepatocytes functional characteristics. The use of organoid models has expanded as they facilitate cell-to-cell or cell-to-ECM communication, mimicking real tissue responses that are key in the regenerative process. One such study modeled primary hepatocyte proliferation through the presence of Epcam (a marker for liver progenitor cells) by suspending 3D hepatocyte organoids in a Matrigel droplet (88). Similarly, Garnier et al. devised a method to prolong the long-term viability of primary hepatocytes through modeling in a 3D organoid model in a cell culture suspension (89). However, after the initial increase in hepatocyte proliferation in the organoid model, both models struggled to maintain long-term growth, as their growth rate declined over time.

One major limitation of organoids is their limited vascularization, which causes necrosis in the dense center of the tissue. They also lack dynamic flow, a feature critical to liver regeneration signaling. Liver organoids and co-culture systems have been primarily used for pharmaceutical research or to study liver injury but have to date not studied liver regeneration in any great depth.

### 3.3 Mechanical and hemodynamic signaling

As mentioned earlier, priming for liver regeneration relies heavily on hemodynamic cues, which are difficult to measure *in vitro* and difficult to temporally control *in vivo*. With the development of microfluidic chip systems, such as Liver-on-a-Chip, these systems provide a platform to model and precisely control fluid flow through perfusion, addressing the limitations associated with static 2D culture systems and organoid culture models. These multicellular chip systems are built to form multicellular organs comprising of hepatocytes, KCs, HSCs, and LSECs, offering a complexity far beyond that of static 2D models. Cells are seeded onto a biocompatible matrix or ECM, then through the establishment of an LSEC conduit, continuous or peristaltic flow of culture media can be used to mimic the vascular dynamics and liver architecture. Perfusion enables the examination of shear stress, mechanical stiffness, oxygen gradient, signaling gradients, and hydrodynamic pressure within the system (90).

Despite these strengths, most liver-on-a-chip systems have been developed primarily to model liver injury, steatosis, or drug toxicity rather than regeneration itself (91–94). Thus, while these systems provide significant advancements over organoids and standard 2D *in vitro* culture systems, their application to regenerative biology remains comparatively underdeveloped.

### 3.4 Structural preservation for regenerative priming

As ECM modeling plays a large role in priming for regeneration signals, approaches such as 3D bioprinting have been utilized to recreate a microenvironment for hepatic lobule structure. By precisely placing collagen type I and hyaluronic acid on scaffolds, researchers can mimic ECM components and support primary hepatocyte growth (95). Another strategy involves the use of decellularized liver models and recellularization with liver stem cells after transplantation in rodent models (96). Decellularized ECM scaffolds have been shown to enhance the growth of differentiated hepatocytes through increased viability and improved functional characteristics of iPSC-derived hepatocytes (97). Notably, liver-derived extracellular matrix is far more effective than artificially derived matrix to support *in vitro* culture of human hepatocytes (98). These methods provide insight into morphological architecture to improve hepatic function, to examine cell-matrix and cell-cell interactions, and to model liver injury and recovery.

Utilizing ECM to build scaffolds has become an important approach to determine how stiffness or mechanosensory stress can influence liver regeneration. Following liver injury, an increase in fibronectin and collagen type I was detected to aid in increasing matrix stiffness to promote hepatocyte proliferation (99). These observations support the value of replicating liver architecture in *ex vivo* systems to model regenerative response to injury.

Precision-cut liver slices are another *ex vivo* approach that researchers have used to understand hepatocyte metabolism (100). Since these liver slices come directly from an *in vivo* model, the liver architecture and structure are conserved while studying cellular metabolism and identifying subcellular targets. However, precision-cut liver slices have not been used to study liver regeneration, but primarily how hepatocytes react to drug toxicity and liver disease through differences in metabolic signatures, such as glucose production (100). Precision-cut liver slices can only be cultured up to 7 days, yet in an *ex vivo* setting, metabolic function is only preserved for 3 days, thus limiting the utilization of this model to study hepatocyte regeneration (101).

Some studies have investigated a combination approach of *in vitro* and *ex vivo* systems. Yanagi et al. combined *in vitro* generated liver buds followed by *ex vivo* 3D bioprinting of liver spheroids to develop new transplantation methods and directly growing liver buds on the host liver (102). However, graft survival deeper than 100 µm required vascularization and stronger regenerative cues. Thus, in future studies, improving how different model systems recapitulate mechanisms of liver regeneration following injury will be key to translating insights into therapies that enhance recovery post-transplantation.

All of these models have provided critical insights into the dynamic components of liver regeneration by focusing on specific aspects. However, their limitations create a broad gap between mechanistic knowledge and clinical implementation.

## 4 Clinical applications of liver regeneration

Strategies to leverage the regenerative capacity of the liver are fundamental to mitigate complications that arise with reductive liver surgery or partial allo-transplantation. Small-for-size syndrome (SFSS) prevents the utility of many organs in the setting of living donor liver transplantation or even split liver deceased donation in some cases. Recently, our group performed the first RAPID (Resection and Partial Liver Transplantation with Delayed Total Hepatectomy) procedure in the US, which is intrinsically reliant on the liver's regenerative capacity by diminishing portal vascular flow to the remnant native liver, bolstering the growth of the grafted liver volume (103). For decades, experimental strategies have been pursued to augment the hepatic regenerative capacity; yet robust methods have not reached the bedside. The consequences of failed regeneration are serious and can result in an increased demand for the growing liver, leading to metabolic and physiological derangements, ultimately leading to poor patient outcomes if the regeneration does not occur in time to compensate for the metabolic demands of the patient. However, with novel technologies now becoming mainstream in liver transplantation, such as normothermic machine perfusion, where the liver function and bile production are supported *ex vivo*, newer perspectives on old questions can be revisited to push these concepts closer to the bedside. Current strategies for clinical interventions reliant on liver regeneration are listed in Table 1.

**Table 1:** Current surgical applications reliant on liver regeneration.

Strategy	Mechanism of hypertrophy	Time course	Advantages	Limitations
Partial Hepatectomy (PHx) (104–107)	Tissue loss inducing cytokine priming	Days to weeks	Established natural regeneration Preservation of native architecture	Risk of post-hepatectomy liver failure Regeneration impaired in cirrhosis or steatosis liver Limited regeneration in FLR size
Portal Vein Embolization (PVE) (108–112)	Percutaneous blockage of blood supply for portal flow redistribution across the future remnant liver (FLR)	4 to 6 weeks	Pre-operative hypertrophy prior to hepatectomy Gradual and controlled hypertrophy Low morbidity	Slow regeneration Weak regenerative stimulus compared to ALPPS
Portal Vein Ligation (PVL) (113–115)	Ligation of portal vein to cause portal flow redistribution across FLR	4 to 8 weeks	Pre-operative hypertrophy prior to hepatectomy Gradual and controlled hypertrophy Low morbidity	Slow response Less predictable hypertrophy than PVE Collateral formation can reduce regenerative success
Liver Venous Deprivation (LVD) (116)	Simultaneous portal vein and hepatic artery embolization across the FLR	3 to 4 weeks	Contralateral hypertrophy Greater FLR regeneration than PVE or PVL	Requires interventional radiology Regeneration slower than ALPPS
Associating Liver Partition and Portal Vein Ligation (ALPPS) (117–120)	Combination of a portal vein ligation and hepatectomy to stimulate flow and growth factors	7 to 10 days	Rapid hypertrophy Increased resectability Short staging interval	Higher rates of morbidity Technical complexity High inflammatory and metabolic stress Limited long term survival data
Salvage Parenchymal Transection (SPT) (121)	Cut tissue similar to ALPPS followed by failed PVE or PVL	6 to 10 days	Reactivate regeneration after failed hypertrophy	Unpredictable regenerative response High complication rates
Live Donor Liver Transplantation (LDLT) (122–127)	Reconstruction of middle hepatic vein (MHV) tributaries, creating shear stress and inflammation	1 to 3 weeks	Expands donor pool Usage of smaller grafts Near-physiological regeneration Regeneration in both donor and recipient	Risk of regeneration impaired in Small-for-Size Syndrome (SFSS) Dependent on portal hyperperfusion balance Donor-recipient mismatch
Split Liver Transplantation (SLT) (128–131)	Increase in portal vein flow and hepatic artery flow, creating shear stress	Variable	Two recipients, decreases waitlist Pediatric and adult grafts regenerate to respective sizes	Uneven regeneration Higher risk of biliary and vascular complications
Resection and Partial Liver Transplantation with Delayed Total Hepatectomy (RAPID) (132–137)	Change in portal pressure and hepatic arterial flow	2 to 3 weeks	Two recipients, decreases waitlist	Complex, multi-stage procedure Limited international experience Risk of graft failure if regeneration is insufficient

### 4.1 Hepatic resection and portal vein embolization/ligation

Removal of a segment, section, or lobe during PHx allows for healthy tissue to regenerate (105). The preserved portion is known as the future liver remnant (FLR), where regeneration is imperative to fully compensate for the body mass of the patient. However, without thoughtful calculation of FLR or control of the portal inflow (which often needs to be augmented to promote growth to the remnant by pre-operative embolization of the diseased segment or lobe), PHx is associated with risk of liver sinusoidal endothelial damage, hemorrhagic necrosis, and post-hepatectomy liver failure (106). These same principles are also applicable to liver transplantation in the setting of living donor transplant or split liver transplantation, for example.

Portal vein embolization (PVE) and the ensuing portal hypertension increase mechanical stretch, pressure, and shear stress on LSECs. The portal vein dilation increases NO and NOS levels, thereby increasing the permeability of growth factors. Additionally, an increase in proinflammatory cytokines, such as IL-6, creates a regenerative priming setting (138). Recent studies have demonstrated that redistribution of portal blood flow following PVE induces an increase in mitogens such as HGF and TGF*α*, contributing to the redistribution of inflammatory cues and initiating liver regeneration (110, 111). The main stimulant for a portal vein ligation (PVL) is ischemia and oxygen deprivation, creating a less invasive procedure and resulting in slower regeneration (139). With PVE and PVL, the liver takes about four to six weeks for sufficient hypertrophy, which could result in insufficient hypertrophy and a prolonged waiting period to treat metastatic liver tumors (119).

### 4.2 Regeneration in living donor liver transplantation (LDLT)

In living donor liver transplantation (LDLT), the majority of the early phase of regeneration occurs within the two weeks following LDLT, characterized by a rapid rate of regeneration (123, 140). LDLT was a method developed in the 1990s due to a severe shortage of viable deceased donor grafts, especially for pediatric patients who often experienced size mismatches (122). The innovation led to an expansion of the donor pool, soon with transplantation conducted in both adults and pediatric patients (124, 125).

Clinical evidence of regeneration has been demonstrated by the increase of pro-inflammatory signals, where TNF*α* levels were higher 24 h after transplantation, and serum IL-6 increased within a week of transplantation (141, 142). By mediating STAT3 signaling, these post-operative signals accelerated hepatocyte proliferation of hepatocytes and increased graft volume in a shortened period.

During LDLT, portal venous flow is also implicated in priming regenerative signals, as it tightly regulates hemodynamic and metabolic dynamics. Venous outflow congestion, which predominantly occurs during the dissection and splitting of the right and left lobes, results in slower regeneration, particularly with the left lateral segment (126). The reduced blood flow perpetuates a reduced oxygen and nutrient supply to the remaining graft, ultimately hindering hepatocyte proliferation.

One major issue that arises from LDLT is the ability to process metabolic cues for regenerative signals. When a donor-recipient size mismatch occurs, the graft is too small to meet metabolic demands, resulting in a metabolic overload and excessive portal hyperperfusion, called Small-for-size syndrome (SFSS) (143). The surplus of both metabolites and shear stress on a smaller hepatic mass increases regenerative signals, leading to excessive damage to hepatocytes (144). The resulting effect dysregulates sinusoidal integrity, resulting in graft dysfunction. SFSS is clinical proof that regeneration must be regulated and not maximized (145). Hypertrophy needs to be a regulated dynamic to ensure regeneration occurs at an optimal rate.

### 4.3 Challenges in impaired regeneration

Across different procedures, these surgical techniques to aid liver regeneration have different time points for inducing hypertrophy. The exact timing for maximal regenerative output is not well defined, nor is whether rapid or controlled hypertrophy is more sustainable. This necessitates understanding the temporal coordination of inflammation, metabolism, and perfusion to further take advantage of the liver's innate regenerative mechanisms.

The change in sinusoidal structure observed in these clinical techniques dramatically alters the hemodynamic effects of the liver graft. Liver regeneration can fail after a hepatic resection due to too much portal vein inflow or high blood pressure (131, 146). Previous studies have found that portal flow over 20 mmHg can cause impaired regeneration and liver dysfunction (147). Long-lasting ischemia or hypoxic conditions can lead to greater ischemia-reperfusion injury (IRI), which can further impair sinusoidal architecture and create hepatocyte islets (148, 149). Although portal pressure and shear stress mechanisms aid LSEC responsiveness to initiate regenerative cues, hemodynamic overload can cause vascular damage and lead to impaired hypertrophy and graft dysfunction. Careful regulation of portal vein inflow and pressure is a necessity to optimize hypertrophy in clinical settings.

Lack of cytokine production, abnormal oxidative stress, or deficiencies in signaling pathways have been proposed to explain impaired liver regeneration (150). Chronic liver disease can lead to a progression towards fibrosis, characterized by an increase in matrix proteins and a decrease in matrix remodeling (78). The inability of the microenvironment to release growth factors or proinflammatory cues leads hepatocytes to senescence, thus impairing liver regeneration. Additionally, in chronic liver diseases, enhanced integrated stress response can result in hepatocyte apoptosis, creating necrotic tissue. Modulating inflammatory cues, matrix remodeling, and growth factor release are necessary to sustain liver regeneration post-operatively (151, 152).

Successful liver regeneration requires extensive metabolic remodeling in lipid handling, glucose utilization, and mitochondrial function (55). Patients with steatosis, characterized by lipid accumulation within hepatocytes, often have higher incidences of complications or mortality after liver resection. If these metabolic demands are not met, it can lead to impaired regeneration that further exacerbates steatosis, fibrosis, or dysfunctional grafts. SFSS grafts are prone to decreased graft survival rates due to insufficiency in meeting metabolic and synthetic demands (153). Thus, careful regulation of the metabolic network is necessary to reduce impaired liver regeneration.

Accelerated hypertrophy does not necessarily equate to full functional recovery, especially in grafts with steatosis or fibrosis. These clinical observations lead to a gap between the induction of liver growth and the ability to restore hepatic function. As transplantation increasingly relies on donors from marginal donors, with increased fat content, for example, or reduced-size grafts, these biological constraints need to be addressed and cannot be overcome by surgical techniques alone. Understanding the biological context underlying impaired regeneration becomes key to recondition grafts and temporally modulate regenerative signaling prior to transplantation. These limitations underscore the need to examine the barriers that limit the translation of regenerative biology into clinical practice.

## 5 Barriers to translation

All liver graft regeneration arises from a highly temporally regulated and context-dependent dynamic that makes it difficult to reproduce or modulate outside the body. In a transplant setting, regeneration does not occur in an idealized physiological context, but within grafts that are exposed to ischemia, inflammation, hypoxia, reperfusion, altered hemodynamics, and metabolic stresses (154). IRI leads to an increase in anaerobic metabolism, cellular depolarization, and mitochondrial dysfunction (155). These grafts are also combined with donor-risk factors, creating a heterogeneous pool of liver grafts with variable regenerative outcomes. As transplantation begins to rely more on marginal donors, these limitations become more pronounced in advancing the inherent ability of liver regeneration in a clinical setting (156). Limitations that pose challenges to recapitulate liver regeneration in transplantation are listed in Table 2.

**Table 2:** Barriers to accelerate liver regeneration in clinical practice.

Barrier	Limitation	Impact on regeneration	Clinical relevance	Potential mitigation
Marginal Donors (156)	Advanced age (>60 years old) liver grafts, donation after circulatory death (DCD), steatosis, fibrosis	Higher burden of IRI Higher inflammatory environment Narrow regenerative window	Higher Early Allograft Dysfunction (EAD), Primary Nonfunction (PNF)	Machine perfusion Metabolic modulation
Steatotic Grafts (151, 152, 157)	Lipotoxicity, mitochondrial stress, risk of developing MASLD	Energy deficit from fatty acids Accumulation of ROS Negative regulation on cell cycle signaling	Delayed graft function, primary non-function	Defatting cocktails Metabolic modulation
Fibrotic Grafts (158–160)	Excessive accumulation of ECM proteins from overaction of HSCs	ECM stiffness Inhibition of DNA synthesis	Silent allograft fibrosis, chronic inflammation	RNA interference (siRNA) to block collagen production
Advanced Age (>60 years old) Grafts (161–164)	Senescent cells secreting inflammatory molecules	Increased inflammatory baseline Increase susceptibility to further tissue damage	Ischemic Reperfusion Injury, graft dysfunction	Targeted immunomodulating therapeutics
Lack of predictive biomarkers (165–167)	Measurement of liver function or injury does not indicate regenerative properties	Regeneration is not being measured	Misclassification of graft	Induce regeneration prior to transplantation Develop regeneration-specific markers

The use of marginal donors introduces expansion of graft usage to address critical organ shortages and reduce transplant waitlists (168). Yet, there are risks associated with decreased graft survival or early allograft dysfunction (EAD) (169). The increase in susceptibility to IRI in marginal grafts leads to a higher inflammatory baseline with production of ROS species, inflammatory signals, and activation of subpopulations of immune cells post-reperfusion (170). Due to heightened inflammation and injury, these livers struggle to recover post-transplant and risk early dysfunction, leading to more cell damage. Marginal grafts are considered those that exhibit advanced age (>60 years old), donation after circulatory death (DCD), hepatic steatosis, and/or split grafts (171). Prolonged warm and cold ischemia time or macrosteatosis in these allografts narrows the regenerative window, creating barriers to translation. The intrinsic regenerative capacity of donor livers can be compromised prior to transplantation based on the graft condition.

### 5.1 Lack of predictive biomarkers

As efforts to aid in promoting regeneration continue, a fundamental question emerges: when can we tell whether liver regeneration will succeed or fail in a graft? The most frequently assessed and studied biomarkers are aspartate aminotransferase (AST), alanine aminotransferase (ALT), and lactate to measure liver function and prevalence of injury (165). AST and lactate are general biomarkers of liver disease, while ALT is a specific biomarker of hepatocyte injury. However, these markers do not necessarily measure regenerative competence but only change dynamically in response to injury or disease progression (167). Regeneration of a liver can be measured post-operatively through CT and MRI imaging, however there is limited information on how these grafts have regenerative capacity (107, 172). Conversely, the full regeneration and an increase in volume of a liver does not guarantee full functional recovery either (173).

These barriers highlight a central challenge in translating liver regeneration biology to clinical practice. Regenerative capacity is highly context-dependent, temporally regulated, and difficult to assess using current viability criteria. Marginal graft quality, intrinsic cellular dysfunction, and the inability to define regenerative responses outside the body limit both prediction and intervention. These challenges highlight the need for a platform that enables controlled manipulation of hemodynamics, metabolism, immune signaling, and molecular pathways while preserving whole-organ architecture for clinical translation.

## 6 Machine perfusion in liver transplantation

The advent of machine perfusion (MP) has created a clinically relevant platform not only for graft preservation, but also for the controlled study and potential induction of liver regeneration *ex vivo* (174). Unlike static cold storage (SCS), which suppresses metabolism to limit injury during transport, MP maintains the liver in a viable and metabolically active state, enabling functional assessment, therapeutic intervention, and dynamic manipulation before transplantation. This has particular importance for marginal donor grafts, which are more susceptible to ischemia-reperfusion injury, early allograft dysfunction, and biliary complications under conventional SCS (175–177). In this context, MP offers clear clinical potential by improving preservation, reducing injury, and expanding safe use of marginal livers.

Beyond preservation, MP also offers a unique research platform for studying regeneration in a human organ setting under *ex vivo* control. In contrast to 2D cultures and organoids, which capture only selected cellular components and lack native architecture, perfused liver grafts preserve intact multicellular structure, vascular anatomy, and physiological cell-cell interactions. This enables interrogation of regenerative responses in a whole-organ context while allowing precise control over flow parameters, oxygenation, nutrients, and therapeutic exposure. Accordingly, MP bridges an important gap between reductionist experimental systems and *in vivo* transplantation models. A major unresolved question, however, is how preservation duration and *ex situ* conditions influence regenerative capacity, particularly because regenerative priming is highly time dependent (178). Thus, machine perfusion should be viewed not only as an emerging preservation strategy but also as a powerful translational model for investigating and potentially directing human liver regeneration before implantation.

### 6.1 Types of machine perfusion

MP preservation simulates physiological conditions *ex vivo* by circulating nutrients and oxygenation while flushing out toxins through the biliary tract. MP can reduce ischemic injury, biliary complications, and early allograft dysfunction compared to SCS (179). In the U.S., the most common form of MP is normothermic machine perfusion (NMP) (82.6%), followed by hypothermic machine perfusion (HMP) (6.7%), and 10% being unknown (out of 1,200 + recorded in OPTN/STAR database) (180).

#### 6.1.1 Hypothermic machine perfusion (HMP)

Fundamentally, all biological and chemical processes are governed by thermal energy. Hence, in lower temperatures, the cellular energy for the production of ATP is lowered, slowing down metabolism and reducing the need for oxygen (181). The structural integrity of the cells is upheld, which leads to a decreased likelihood of EAD (182). HMP provides nutrients and access for the removal of metabolic by-products or toxins, thus improving hepatocellular function and increasing tissue viability as compared to SCS (183). However, in HMP, the perfusate is not actively oxygenated, leading to the development of hypothermic oxygenated machine perfusion (HOPE) or dual-HOPE (dHOPE) (184, 185). The introduction of oxygenation has been associated with a decrease in biliary complications and enhanced aerobic metabolism, which has been shown to be associated with improved graft survival and reduced EAD (186).

HMP itself still has its own limitations due to the hypothermic conditions, causing hepatocyte metabolism to remain suppressed (187). Additionally, bile production is not supported in this form of MP. Most importantly, for all of these hypothermic MPs, the cold storage preservation prevents real-time functional viability assessment, leading to limited interventions during perfusion (188).

#### 6.1.2 Normothermic machine perfusion (NMP)

NMP was developed as a method to prolong organ preservation and to assess real-time organ viability *ex vivo* prior to transplantation (189). NMP requires perfusion through the portal vein and hepatic artery to maintain liver function, such as maintaining pH, bile production, lactate clearance, and glucose metabolism (190). NMP requires oxygenated blood-based solution as a perfusate at body temperature (35 °C to 38 °C), mimicking physiological conditions. By maintaining normothermic conditions, NMP aims to reduce the impact of IRI by preserving metabolic and biliary function. In several clinical trials, it was found that NMP extended preservation time, improved patient and graft survival, and maintained metabolic hepatocyte and biliary function (191–193).

Progress with NMP has far advanced by increasing long-term viability through the adjustment of perfusate with nutrients and oxygenation, where researchers have marginal allografts on NMP for up to 7 days, even splitting whole liver grafts on a pump to increase organ availability (194, 195). NMP has been utilized in a pig model of split liver transplantation, which improved post-operative outcomes compared to those split under SCS (196). Several European groups have demonstrated successful liver transplantation following prolonged NMP (48 to 68 h). Together these studies support the potential for prolonged ex-vivo perfusion, a factor that could increase the therapeutic window to induce repair and regenerative cues (197, 198). By preserving the liver in its physiological conditions, a unique opportunity for therapeutic intervention during *ex vivo* perfusion is possible through the addition of substances into the perfusate. Thus, a major limitation of MP-based regenerative therapy is the mismatch between the time scale of clinical perfusion and the biological time course of liver regeneration. Hepatocyte priming begins within minutes to hours after injury, but cell-cycle progression, tissue mass restoration, vascular remodeling, and functional recovery unfold over days to weeks. Therefore, conventional 6–12-hour NMP should not be viewed as a platform to complete regeneration *ex vivo*. Instead, its immediate translational value lies in preserving regenerative competence, reversing injury-associated barriers, and initiating early priming signals before implantation. Longer-duration NMP platforms may expand this window and allow assessment of later proliferative endpoints, but this remains an emerging area requiring further validation.

#### 6.1.3 Alternative machine perfusion systems

Other MP methods include subnormothermic machine perfusion (SNMP), normothermic regional perfusion (NRP), and controlled oxygenated rewarming (COR). Subnormothermic machine perfusion is typically performed at approximately 21 °C, a temperature intended to partially restore metabolic activity while avoiding the full oxygen and energetic demands of normothermic perfusion (199, 200). Graft or patient survival data are limited for NRP, hence this technique needs more information on human clinical trials, as it is limited to animal models. NRP is the *in situ* perfusion of a liver from a DCD, thus maintaining normal body temperature to minimize damage and “resuscitate” the liver (201). NRP raises ethical concerns about defining death of a donor or legal complications and requires strict protocols to avoid brain reperfusion and to maintain death determination standards. Lastly, COR focuses on incremental rewarming from cold-stored liver with oxygenated perfusion (202). This technique avoids sudden thermal changes and oxidative stress during the reperfusion process, thus protecting the liver from IRI damage. However, there is still limited data on this technique.

By preserving liver grafts on MP, a unique opportunity to induce regeneration through the addition of therapeutics in perfusates creates a possibility to overcome limitations associated with ECD grafts. Provided with the knowledge behind early regenerative signals, MP opens a potential to model *ex vivo* regeneration that has only been previously captured *in vivo*. For organs that require more care prior to transplantation or do not pass the viability assessment, machine perfusion offers a platform to repair the liver by delivering regenerative agents through the perfusate (203). This opens an opportunity to repair and recondition the liver through immunomodulation or other treatment options in the future.

### 6.2 Machine perfusion as a platform for regenerative competence, priming, and augmentation

**Figure 2:**
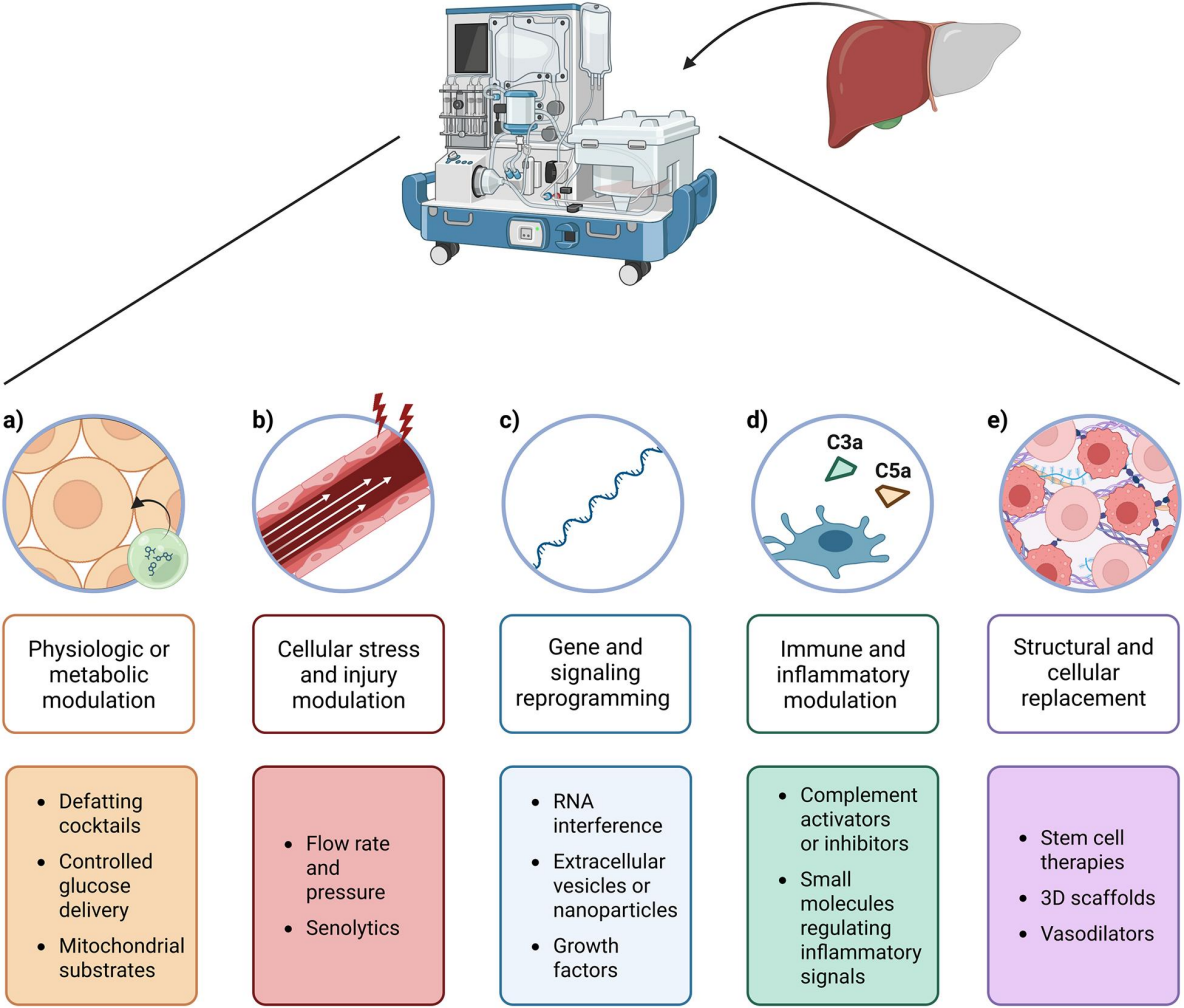
*Ex vivo* liver perfusion as a platform for regenerative priming of liver grafts. Schematic illustrating how *ex vivo* liver perfusion (EVLP) could be utilized to condition donor livers prior to transplantation to enhance their regenerative capacity, particularly in small-for-size grafts. The perfusion circuit maintains the liver in a metabolically active state, enabling targeted delivery of therapeutic interventions during preservation. Five mechanistic domains through which EVLP may promote regeneration are highlighted. **(a)** Physiologic and metabolic modulation: optimization of oxygenation and substrate delivery, including defatting strategies, controlled glucose delivery, and mitochondrial support to restore hepatocellular energy metabolism. **(b)** Cellular stress modulation: administration of cytoprotective agents to limit ischemia–reperfusion injury, oxidative stress, and endothelial dysfunction. **(c)** Gene and signaling reprogramming: delivery of RNA therapeutics, extracellular vesicles, viral vectors, or growth factors to activate regenerative pathways such as IL-6/STAT3, HGF/c-MET, Wnt/*β*-catenin, and Hippo–YAP/TAZ. **(d)** Immune and inflammatory modulation: attenuation of innate immune activation and complement signaling to reduce inflammatory injury**. (e)** Structural or cellular replacement: cell-based therapies or biomaterial scaffolds that support tissue repair and microvascular stability. Together, these strategies position EVLP as a pre-implantation regenerative conditioning platform, transforming organ preservation into an opportunity to enhance graft recovery and regeneration. Created in Biorender.

By developing viable treatments for the liver on a machine perfusion pump, it expands the possibility to maintain the liver for long-term usage, split the liver into small-for-size grafts and promote regeneration. A key distinction, however, must be made between interventions that improve graft preservation and those that directly augment regeneration. Current MP-based interventions are designed to reduce ischemia–reperfusion injury, improve mitochondrial function, restore bile production, or enhance overall viability (summarized in Figure 2). These effects may preserve or restore regenerative competence, but to date they have not been shown to and do not necessarily induce hepatocyte cell-cycle entry, proliferative expansion, or tissue regrowth. Therefore, MP should not be viewed as a regenerative therapy in its current clinical form. Rather, it represents a uniquely powerful platform through which regenerative biology can be studied, preserved, and potentially through scientific innovation directed before implantation.

Here, we have distinguished between three conceptually distinct MP-based strategies: regenerative-permissive strategies, which reduce barriers to regeneration by limiting injury or restoring metabolic and vascular competence; regenerative-priming strategies, which activate early cytokine, complement, metabolic, mechanosensory, or growth factor pathways that license hepatocyte proliferation; and regenerative-replacement strategies. This framework allows MP to be considered not merely as a preservation technology, but as a controllable *ex vivo* platform for temporally and spatially manipulating the early events that determine regenerative capacity. However, major unresolved questions still remain in how preservation duration and ex situ conditions influence regenerative potential, particularly because regenerative priming is highly time dependent. Thus, MP may be best understood as a translational bridge between graft reconditioning and therapeutic regenerative priming, rather than as a completed regenerative intervention itself.

#### 6.2.1 Regenerative-permissive interventions

Current efforts of metabolic modulation focus on repair and organ assessment for livers on MP, however given the role of metabolic changes as a regenerative cue to hepatocyte proliferation (see Section 2.6), altering metabolic changes may open a possibility for regenerative medicine on MP. Defatting cocktails have primarily been used for the purposes of repairing steatotic livers, as they are associated with a higher rate of EAD (204). Defatting cocktails typically include mixtures of compounds that will enhance fatty acid oxidation, autophagy, and secretion of low-density lipoproteins (205, 206). Such studies have led to improving hepatocellular function and decreased expression of IRI markers (207). Beyond defatting and improving hepatocellular function, metabolic modulation, such as mitochondrial substrates or redox-balancing agents, could potentially be used to restore ATP substrates and improve mitochondrial function during NMP.

Controlled glucose delivery is also an open avenue to explore insulin-responsive signaling pathways that are suppressed during cold ischemia (208). The restoration of cellular energetics can serve as a cue for regenerative priming and support early regenerative responses to influence hepatocyte cell cycle re-entry for the improvement of post-transplant graft viability.

#### 6.2.2 Regenerative priming interventions

Senolytics involve a group of pharmacological agents responsible for inducing apoptosis in senescent cells. Although to this date, no senolytics have been delivered through NMP (209). There has been evidence that senolytics provide mitochondrial protective effects and restore liver regeneration in mice (210, 211). NMP as a platform provides a space for targeted clearance through waste collection systems without creating systemic toxicity. Thus, removal of these senescence cells could prevent senescence-associated phenotypes that inhibit regeneration, creating a primed regenerative microenvironment prior to transplantation.

To maintain physiological conditions, typical pressures of NMP range from 60 to 70 mmHg in the hepatic artery and 8–11 mmHg in the portal vein, resulting in a flow rate of 0.2–0.3 L/min and 0.8–1.25 L/min, respectively (212). Shear stress is recommended to be minimized on NMP to allow for homogeneous perfusion across a whole liver and prevent endothelial injury (213). Recent work in split liver and small graft perfusion has created an unprecedented opportunity to explore mechanosensory stimulation of regeneration. In a 1-week long preservation of split human livers on NMP, smaller left lateral segment grafts exhibited higher perfusion flow normalized to tissue mass compared to the extended right grafts (195). Given the link established between shear stress and regeneration, such as LSEC-mediated NO production and HSC-mediated growth factor release, smaller grafts may play an intrinsic biomechanical stimulation for regenerative priming during NMP (214). To this date, there has been no deliberate manipulation of flow, pulsatility, and shear stress on NMP as a process to aid liver regeneration, creating an open avenue for a non-pharmacologic strategy to prime regenerative signaling.

As IL-6 and TNF*α* play predominantly large roles in the priming phase, modulating the release of cytokines could create a regulated inflammatory environment to induce pro-regenerative signaling. One research group found that the therapeutic effects of enkephalin, a proinflammatory delta opioid agonist upregulating IL-6 and TNF*α*, in NMP rat livers were driven by alteration of cytokine signaling and modulating inflammatory response during IRI (215). This study highlights an avenue to recalibrate inflammatory signaling towards regenerative priming during NMP. The reduction of IRI and improvement of hepatocyte function enable entry into regenerative programming that can be temporally regulated on NMP.

Given complement fragments, C3a and C5a, are required for effective liver regeneration following a PHx, NMP opens an avenue to controlled complement modulation, which has yet to be explored (21). In transplantation, complement activation is seen as an injurious process due to its role in IRI (216). Since complement activation occurs locally in KCs and hepatocytes, NMP provides a unique platform to augment or selectively activate regenerative capabilities while avoiding terminally mediated complement injury (216). Through NMP, the introduction of complement factors provides a unique temporal method to complement-mediated regenerative priming and decouples from the adverse effects of systemic inflammatory injury prior to transplantation.

#### 6.2.3 Regenerative replacement or reconstructive strategies

In contrast to regenerative-permissive approaches that reduce injury and regenerative-priming approaches that activate endogenous signaling pathways, regenerative replacement or reconstructive strategies aim to reprogram, supplement, or rebuild the cellular and structural components required for liver repair. These approaches include RNA-based pathway modulation, extracellular vesicle delivery, growth factor supplementation, stromal or progenitor cell therapy, hepatocyte transplantation, and vascular niche repair. Although most remain exploratory in the context of machine perfusion, they represent an important frontier in translating liver regenerative biology toward graft-directed therapy.

RNA interference has emerged as a potential strategy to transiently modify gene expression during *ex vivo* perfusion (217). Current applications have largely focused on graft protection or repair rather than direct regenerative induction. For example, antisense oligonucleotide delivery has been used to induce resistance to hepatitis C virus in NMP-perfused livers, demonstrating the feasibility of nucleic-acid-based intervention during perfusion (218). In addition, studies showing that siRNA can be taken up by hepatocytes *ex vivo* and alter cellular signaling support the possibility that MP could be used to tune pathways relevant to regenerative competence (219, 220). In principle, transient RNA-based modulation could be used to suppress anti-regenerative programs, enhance hepatocyte responsiveness to mitogens, or recalibrate inflammatory, metabolic, and stress-response pathways that shape regenerative priming. However, these strategies have not yet been directly validated as methods to induce liver regeneration during MP (150, 209).

Extracellular vesicles provide a complementary approach for signal-level reprogramming. Unlike direct RNA delivery, extracellular vesicles can carry coordinated cargoes of miRNAs, proteins, and lipids, thereby more closely mimicking endogenous intercellular communication (221). Stem cell-derived extracellular vesicles may also provide partial cell-type specificity and could be engineered to deliver regenerative cues to hepatocytes, LSECs, KCs or HSCs. Prior work showing that extracellular vesicles are absorbed by hepatocytes during NMP within hours of perfusion supports the feasibility of this approach. However, current evidence primarily demonstrates effects on ischemic and hypoxic injury rather than direct induction of proliferative regeneration. Thus, EV-based strategies may be best viewed as an emerging platform for regenerative priming rather than established regenerative replacement.

Growth factor delivery represents a more direct strategy to recreate early regenerative signaling gradients. HGF, EGF, and VEGF are central mediators of hepatocyte proliferation, endothelial–parenchymal crosstalk, angiogenesis, and tissue remodeling (213). Supplementation of these factors during MP could theoretically reproduce aspects of the early post-hepatectomy milieu and promote hepatocyte cell-cycle competence. However, the regenerative effects of growth factor delivery are likely to be highly dependent on dose, timing, spatial distribution, and graft condition. Uncontrolled exposure could also promote maladaptive proliferation, endothelial dysfunction, or imbalanced angiogenic signaling. Therefore, although growth factor modulation is conceptually attractive, it requires careful temporal and mechanistic validation before clinical translation.

Cell-based strategies provide another reconstructive approach. Mesenchymal stromal cells have been delivered during NMP in preclinical models and are thought to reduce ischemia–reperfusion injury, modulate inflammation, and secrete hepatotrophic factors such as HGF and VEGF (222–225). Multipotent adult progenitor cells delivered through the hepatic artery have also been associated with cytoprotection, extracellular matrix remodeling, and activation of cell-cycle-related pathways in perfused livers. These findings suggest that cell therapy during MP may condition the regenerative niche. However, the benefits of these therapies are more likely mediated by paracrine signaling than by durable engraftment or tissue replacement. Therefore, MP may amplify cell-derived regenerative signals, but whether it enables true cellular reconstruction remains unresolved.

Hepatocyte transplantation is the most direct cellular replacement strategy and has been explored for metabolic liver diseases and acute liver failure (226). By introducing functional hepatocytes, this approach seeks to restore liver function through cellular engraftment and proliferation (227). However, clinical effects have generally been limited by poor engraftment, immune clearance, short-lived benefit, and the requirement for a permissive regenerative microenvironment (228). The ability to deliver hepatocytes through the perfusion circuit during split-liver MP or small-for-size graft conditioning is conceptually appealing but remains unproven (229). Future studies will need to determine whether MP can improve cell distribution, engraftment, survival, and functional integration.

Reconstructing the vascular and endothelial niche may be essential for regenerative success. LSECs regulate nitric oxide production, angiocrine signaling, HGF and Wnt pathway activation, and the response to mechanosensory forces. Strategies that protect LSECs during cold storage or restore hepatic arterial and portal flow may therefore preserve the regenerative microenvironment. Vasodilators such as BQ123, verapamil, epoprostenol, and prostaglandin I2 have been used to reduce endothelial injury and improve hepatic arterial flow, but most studies have evaluated short-term graft injury rather than regeneration-specific endpoints (230). Thus, endothelial protection should be considered a regenerative-permissive or niche-restorative strategy, rather than definitive evidence of regenerative augmentation.

Finally, the conversion of bioartificial liver (BAL) support systems with MPs could provide synthetic support for *ex vivo* liver function (231). BALs provide a whole systems approach to stimulate *in vivo* liver function through a mechanical filtration circuit with an *ex vivo* cellular source of healthy hepatocytes (232). The basic circuit involves the patient's blood drawn out to run through a BAL device, where plasma is separated from the blood to circulate with hepatocytes in a bioreactor that removes toxins before the blood and plasma are returned to the patient. However, interaction of *ex vivo* hepatocytes with patient plasma does not elicit a regenerative response because an interface with native tissue architecture is necessary to reconstruct, remodel, or regenerate the liver. While BALs provide a potential to improve synthetic function in MPs, additional progress in improving the BAL technology beyond preclinical trials is required before implementation (229).

Together, these approaches position MP as a potential interface between graft preservation and regenerative reconstruction. A major advantage of MP is that it maintains vascular access, physiologic flow, multicellular architecture, and controlled therapeutic exposure, thus positioning it as a platform that may enable delivery of RNA therapeutics, extracellular vesicles, growth factors, stromal cells, hepatocytes, or endothelial-supportive agents directly to the graft before implantation. However, these strategies remain largely preclinical, and their regenerative effects must be distinguished from improvements in viability or injury reduction. Future studies should incorporate regeneration-specific endpoints, including hepatocyte cell-cycle entry, proliferative expansion, angiocrine signaling, matrix remodeling, and post-transplant functional recovery.

### 6.3 Implications for *ex vivo* perfusion (a clinical paradigm)

To increase the organ supply, a Split Liver Transplantation (SLT) was introduced in 1988 by Rudolf Pichlmayr, primarily for pediatric patients (128). Improved outcomes of treatment were achieved, yet, initially, when applied to small adult recipients, the patient and graft survival were still lower compared to a whole liver transplantation (129, 130). Split grafts are often used in the ideal donor candidates and are sparingly used in the US given donor populations. LDLT utilizes split techniques in a live setting, often has excellent outcomes, yet is relatively uncommon in the US compared to international programs. Particular disease processes rely on the use of living donors, however, they have not yet reached mainstream in the US. For example, colorectal metastasis to the liver was long considered a contraindication to liver transplant. Yet the Scandinavian SECA-I and SECA-II trials proved that 5-year patient survival can reach up to 80% in selected patients where the primary colon cancer is removed, and the tumor burden is confined to the liver without aggressive tumor biology. This is compared to a meager 10% survival with standard of care systemic chemotherapy (233, 234). However, not many centers in the US offer protocols for transplant in colorectal liver metastasis (CRLM), which can limit options for these patients who only get a model for end-stage liver disease (MELD) exception of 15. Patients are encouraged to seek a living donor, which is often a barrier to transplant.

To address this unmet need, a two-step liver transplantation technique was introduced by Line, et al. in 2015, RAPID for colorectal liver metastases (134). The RAPID procedure classically consists of transplanting a left lateral segment graft from a live donor into a patient with CRLM while removing the left cancerous lobe and ligating the right portal vein. The segment is insufficient to maintain the physiology for the recipient, so the cancerous but functional right lobe is left in place to augment the function and support the patient until the graft grows over a 10–14 day period, at which time the patient is taken back to the operating room for a completion hepatectomy, completing the oncologic operation (134). RAPID focuses on the regeneration of the liver as a prominent role, then restoration of function followed.

In Europe, clinical trials of RAPID have been published for patients with noncirrhotic liver disease and hepatocellular carcinoma (136, 137). In fact, multiple studies in Europe have found positive outcomes of patients and graft survival through the RAPID technique within an average of three weeks of residual hepatectomy (132, 133, 135–137, 235, 236). However, all of these procedures rely on determining splitting of the liver on site, whether *in situ* or on a machine perfusion pump. In other words, using machine perfusion is viewed as an optional case.

Our group performed the first case of RAPID in the United States in 2023, with a completion hepatectomy conducted two weeks after initial graft transplantation, with the use of NMP to preserve the graft (103). A whole allograft was procured from a DCD donor and split on an NMP. Adding NMP helps extend the graft preservation while providing real-time viability assessment to ensure both grafts remain viable throughout the entire split procedure. This technique overall saved both patients from end-stage liver disease and has the potential to expand the donor pool for select patients.

The RAPID experience is still underutilized but offers regenerative medicine applications in the future (135). As RAPID prioritizes taking advantage of the liver's innate regenerative mechanisms, the graft on NMP can be temporarily boosted by adding different modulators to the perfusate to support metabolic function, detoxification, and hepatocyte proliferation.

Some challenges to consider are that the smaller portion of the liver graft is prone to SFSS. SFSS is caused by increased portal pressure and hyperperfusion, which can cause damage to the microvasculature in liver sinusoids, thus causing failed regeneration and increasing EAD (237). Hence, there is a higher early post-transplant risk compared to a whole liver transplantation, so it requires more careful monitoring in the post-operative procedure, as the risk of the graft decreases over time (238). Another circumstance to consider is determining the consensus criteria that determine whether a liver is suitable for splitting.

## 7 Conclusion

In summary, liver regeneration is a tightly coordinated, multicellular process governed by the integration of inflammatory priming, complement activation, mechanosensory signaling, extracellular matrix remodeling, metabolic reprogramming, and growth factor–driven proliferation. Although decades of *in vivo* and *in vitro* work have defined many of the pathways that underlie regenerative competence, translating these findings into clinically actionable therapies has remained limited by the complexity, temporality, and context dependence of the response. Machine perfusion now offers a compelling opportunity to bridge this gap by providing a controllable *ex vivo* platform in which whole-organ architecture is preserved while regenerative cues can be measured, manipulated, and therapeutically augmented before implantation. As transplantation increasingly depends on marginal, steatotic, aged, and reduced-size grafts, the ability to recondition and prime the liver for regeneration *ex situ* may become central to improving graft utilization and post-transplant outcomes. The next phase of the field will require integration of mechanistic biology with translational perfusion platforms to define when, how, and in whom regeneration can be safely accelerated, ultimately moving liver regeneration from a passive biological phenomenon to an actively engineerable therapeutic strategy.
